# Therapeutic International Normalized Ratio Monitoring

**DOI:** 10.4274/tjh.2016.0467

**Published:** 2017-03-01

**Authors:** Beuy Joob, Viroj Wiwanitkit

**Affiliations:** 1 Sanitation 1 Medical Academic Center, Bangkok, Thailand; 2 Hainan Medical University, Haikou, China

**Keywords:** monitoring, International normalized ratio, Hemostasis

## TO THE EDITOR,

The report on “Warfarin dosing and time required to reach therapeutic international normalized ratio in patients with hypercoagulable conditions” was very interesting [1]. Kahlon et al. concluded that “Patients with hypercoagulable conditions require approximately 10 mg of additional total warfarin dose and also require, on average, 2 extra days to reach therapeutic international normalized ratio (INR) as compared to controls.” The big concern in this report regards the technique used for INR measurement. Kahlon et al. did not mention this and might not have noted the problem of measurement of INR in the follow-up of the patient. The quality control of the measurement is very important and measurements from different laboratory techniques and settings can be a factor leading to error in laboratory results [2,3]. It is noted that the local calibration in correcting the variability in INR determination and the difference between batches has to be controlled [4].
